# Impact of heart rate variability, a marker for cardiac health, on lupus disease activity

**DOI:** 10.1186/s13075-016-1087-x

**Published:** 2016-09-02

**Authors:** Aikaterini Thanou, Stavros Stavrakis, John W. Dyer, Melissa E. Munroe, Judith A. James, Joan T. Merrill

**Affiliations:** 1 Arthritis and Clinical Immunology Research Program, Oklahoma Medical Research Foundation, 825 N.E. 13th Street, Oklahoma City, OK 73104 USA; 2 Heart Rhythm Institute, University of Oklahoma Health Sciences Center, 1200 Mark Everett Drive, TCH 6E103, Oklahoma City, OK 73104 USA; 3 Departments of Medicine and Pathology, University of Oklahoma Health Sciences Center, Oklahoma City, OK USA

**Keywords:** Systemic lupus erythematosus, Disease activity, Heart rate variability, Cytokines

## Abstract

**Background:**

Decreased heart rate variability (HRV) is associated with adverse outcomes in cardiovascular diseases and has been observed in patients with systemic lupus erythematosus (SLE). We examined the relationship of HRV with SLE disease activity and selected cytokine pathways.

**Methods:**

Fifty-three patients from the Oklahoma Lupus Cohort were evaluated at two visits each. Clinical assessments included the Systemic Lupus Erythematosus Disease Activity Index (SLEDAI), British Isles Lupus Assessment Group (BILAG) index, physician global assessment (PGA), and Safety of Estrogens in Lupus Erythematosus National Assessment-SLEDAI Flare Index. HRV was assessed with a 5-minute electrocardiogram, and the following HRV parameters were calculated: square root of the mean of the squares of differences between adjacent NN intervals (RMSSD), percentage of pairs of adjacent NN intervals differing by more than 50 milliseconds (pNN50), high-frequency power (HF power), and low frequency to high frequency (LF/HF) ratio, which reflects sympathetic/vagal balance. Plasma cytokine levels were measured with a multiplex, bead-based immunoassay. Serum B lymphocyte stimulator (BLyS) and a proliferation-inducing ligand (APRIL) were measured with an enzyme-linked immunosorbent assay. Linear regression analysis was applied.

**Results:**

Baseline HRV (pNN50, HF power, LF/HF ratio) was inversely related to disease activity (BILAG, PGA) and flare. Changes in RMSSD between visits were inversely related to changes in SLEDAI (*p* = 0.007). Age, caffeine, tobacco and medication use had no impact on HRV. Plasma soluble tumor necrosis factor receptor II (sTNFRII) and monokine induced by interferon gamma (MIG) were inversely related with all baseline measures of HRV (*p* = 0.039 to <0.001). Plasma stem cell factor (SCF), interleukin (IL)-1 receptor antagonist (IL-1RA), and IL-15 showed similar inverse relationships with baseline HRV, and weaker trends were observed for interferon (IFN)-α, interferon gamma-induced protein (IP)-10, and serum BLyS. Changes in the LF/HF ratio between visits were also associated with changes in sTNFRII (*p* = 0.021), MIG (*p* = 0.003), IFN-α (*p* = 0.012), SCF (*p* = 0.001), IL-1RA (*p* = 0.023), and IL-15 (*p* = 0.010). On the basis of multivariate linear regression, MIG was an independent predictor of baseline HRV after adjusting for plasma IL-1RA, SCF, IFN-α, IP-10, and serum BLyS. In a similar model, the sTNFRII impact remained significant after adjusting for the same variables.

**Conclusions:**

Impaired HRV, particularly the LF/HF ratio, is associated with lupus disease activity and several cytokines related to IFN type II and TNF pathways. The strongest association was with MIG and sTNFRII, expanding previous immune connections of vagal signaling.

**Electronic supplementary material:**

The online version of this article (doi:10.1186/s13075-016-1087-x) contains supplementary material, which is available to authorized users.

## Background

Neural influence on immune function can been profound, raising the question of neuroimmune modulation in chronic inflammatory conditions such as systemic lupus erythematosus (SLE) [1]. Autonomic dysfunction is well documented in SLE, with increased sympathetic and decreased parasympathetic activity reported by different studies [2–9]. Such autonomic aberrations can be the result of inflammatory cytokines acting on the central nervous system and may reciprocally modulate inflammatory responses in the periphery, exerting a pathogenic role in chronic inflammation [10].

Both sympathetic and parasympathetic pathways are relevant in acute and chronic inflammation, based on in vitro studies and animal models. Upon initiation of an acute inflammatory process, the body adopts a nonspecific “inflammatory configuration” with increased systemic sympathetic activity and hypothalamic-pituitary-adrenal axis activation [11]. At the same time, sympathetic neurotransmitters act directly on immune cells, promoting primarily anti-inflammatory mechanisms at the tissue level. As inflammation becomes chronic, tissue regulation is uncoupled from central influence by repulsion of sympathetic nerve fibers that promotes an inflammatory phenotype [11]. Signaling through the vagus nerve has strong anti-inflammatory effects in tissue-specific and systemic inflammation [1]. Efferent potentials from the brainstem inhibit cytokine production in the spleen through a complex pathway that also includes the splanchnic sympathetic nerves [12]. Recent understanding of this pathway suggests a non-neural link between the vagus nerve and the spleen, possibly through vagus-induced mobilization of peripheral lymphocyte pools that subsequently accumulate in the spleen [12].

Autonomic activity can be measured through a range of techniques that include clinical cardiovascular reflex testing and heart rate variability (HRV) [13]. HRV is a noninvasive tool that measures cyclical fluctuations in resting heart rate to indirectly assess cardiac parasympathetic and sympathetic influences. Although vagal tone is considered organ-specific, HRV is often used as a surrogate for systemic vagal output. Decreased HRV is linked to increased cardiovascular morbidity and mortality, as well as an increased prevalence of heart failure and arrhythmias after myocardial infarction [14, 15]. Measures of HRV have also been inversely correlated with serum inflammatory biomarkers (C-reactive protein, interleukin [IL]-6) in healthy individuals as well as in those with cardiovascular diseases [15].

We hypothesized that aberrant autonomic function reflected by HRV is related to disease activity in SLE, and we examined this hypothesis cross-sectionally and longitudinally in a cohort of well-characterized patients with active disease who were receiving standard-of-care treatments. We further explored associations of HRV with a large panel of cytokines, including several pertinent to SLE.

## Methods

We conducted a prospective cohort study at the Department of Arthritis and Clinical Immunology of the Oklahoma Medical Research Foundation (OMRF) as a project of the Oklahoma Lupus Cohort, a longitudinal study that includes 610 patients who meet the 1997 SLE classification criteria [16]. The protocol was approved by the OMRF Institutional Review Board (IRB). All patients underwent informed consent procedures consistent with the governance of the OMRF IRB and the Declaration of Helsinki. Fifty-three consecutive patients with SLE from the Oklahoma Lupus Cohort with active disease at baseline were examined at two time points while standard of care for lupus was rendered. Active disease at baseline was defined as Systemic Lupus Erythematosus Disease Activity Index (SLEDAI) of at least 4 points or at least one B score on the British Isles Lupus Assessment Group (BILAG) index [17]. Patients were evaluated using the hybrid SLEDAI, which is identical to the Safety of Estrogens in Lupus National Assessment (SELENA)-SLEDAI [18, 19] except for the proteinuria definition from the SLEDAI 2000 (SLEDAI-2K) [20, 21]. Presence of flare at each visit was determined by the SELENA-SLEDAI Flare Index (SFI) with the physician global assessment (PGA) [18, 19]. Laboratory tests at each visit included complete blood counts, a comprehensive metabolic panel, urinalysis and C3 and C4 complement levels. In addition to these routine laboratory assessments, serum B lymphocyte stimulator (BLyS) and a proliferation-inducing ligand (APRIL), as well as a panel of plasma cytokines were measured at each visit by enzyme-linked immunosorbent assay and by xMAP® multiplex bead-based immunoassay (eBioscience/Affymetrix, San Diego, California, USA) respectively. The following plasma cytokines were included in the panel: interferon (IFN)-γ, IL-12p70, IL-2, IL-1α, IL-1β, interleukin (IL)-1 receptor antagonist (IL-1RA), IL-15, IL-17A, IL-21, tumor necrosis factor (TNF)-α, soluble tumor necrosis factor receptors I and II (sTNFRI/II), soluble CD40L (sCD40L), BLyS, APRIL, transforming growth factor (TGF)-β, IL-10, IFN-α, interferon gamma-induced protein 10 (IP-10), macrophage inflammatory protein 1 alpha (MIP-1α), monokine induced by interferon gamma (MIG), IL-8, plasminogen activator inhibitor 1 (PAI-1), stem cell factor (SCF), and resistin (see Table S1 in Additional file 1).

Medical records were reviewed and a questionnaire about variables that might affect HRV was completed at each clinic visit. Potentially confounding variables that were evaluated included any heart rhythm other than sinus, cardiomyopathy (left ventricular ejection fraction <40 %), recent (<1 year) myocardial infarction or unstable angina, heart failure (New York Heart Association class III or IV), uncontrolled hypertension (systolic blood pressure >160 mmHg on at least three antihypertensive medications), mitral valve prolapse (other than mild), recurrent vasovagal syncopal episodes, unilateral or bilateral vagotomy, concomitant use of medications potentially affecting autonomic function (e.g., anticholinergic medications and β-blockers), significant neurologic disorder (e.g., Parkinson’s disease, multiple sclerosis, Guillain-Barré syndrome), major untreated depression or psychosis, obstructive sleep apnea, asthma or chronic obstructive pulmonary disease not controlled by medications, any other disease causing clinically significant dyspnea at the time of assessment, and uncontrolled insulin- or non-insulin-dependent diabetes. Patients were also questioned about recent exercise and tobacco use (preceding 12 hours), any caffeine consumed in the 6 hours prior to testing, and alcohol consumed within 12 hours of testing.

A 5 minute electrocardiogram (ECG) tracing was recorded at each visit using a modification of the commercially available AliveCor iPhone ECG device (AliveCor, San Francisco, CA, USA). ECG electrode patches were attached to the right and left forearms and connected to the device with a custom-made attachment to enable stable ECG recording (lead I) over 5 minutes. Recordings were transmitted online to a Health Insurance Portability and Accountability Act (HIPAA)-compliant server with validated encryption technology. ECG results were processed blindly, and the following HRV parameters were obtained by time domain analysis: (1) square root of the mean of the squares of differences between adjacent NN intervals (RMSSD) and (2) percentage of pairs of adjacent NN intervals differing by more than 50 milliseconds (pNN50). The following HRV parameters were obtained by frequency domain analysis: (1) low-frequency power (LF power), (2) high-frequency power (HF power), and (3) the LF/HF ratio [22]. Both short-duration (5 minutes) and long-duration (24 hours) ECG recordings are acceptable options for measuring HRV [22]. Nonetheless, frequency domain analysis is the preferred method when short-duration recordings are examined [22]. Normative values of these HRV measures have yet to be established, and HRV testing in isolation cannot differentiate increased sympathetic from decreased parasympathetic signaling. However, HF power overall reflects activity of the efferent vagus nerve to the heart, and LF power is considered a measure of sympathetic activity, whereas the ratio of low to high frequency (LF/HF) reflects sympathetic to parasympathetic balance [13]. Reduced RMSSD and pNN50 are also indicative of parasympathetic insufficiency.

### Statistical analysis

Categorical variables were expressed as percentages and continuous variables as median and interquartile range (IQR). Paired *t* tests or Wilcoxon signed-rank tests were used to evaluate changes in disease activity and HRV between visits. A chi-square test with the Yates correction was used to compare immune-suppressive medications and parameters affecting HRV between visits. We used linear regression to evaluate cross-sectional associations of HRV and SLE disease activity, cytokines, medications, and tobacco, caffeine, and alcohol exposure. The natural logarithm of each cytokine was employed to achieve normality. Factors significant in univariate analysis (*p* < 0.1) were entered into multivariate models. To avoid overfitting, no more than one parameter for each ten subjects was examined in the multivariate regression models, which allows for a nonbiased calculation of the regression coefficients [23]. Changes in HRV between baseline and follow-up were also examined as predictors of changes in disease activity by linear regression. The same approach was used to evaluate whether changes in HRV between baseline and follow-up could be predicted by changes in cytokines. In these models, only cytokines found to be significantly associated with HRV in the baseline regression analysis were included. Statistical significance was declared at *p* < 0.05. SigmaPlot 12.5 software (Systat Software, San Jose, CA, USA) was used for all statistical analyses.

## Results

### Characteristics of the study population

Fifty-two female and one male patient were studied. Their median age at baseline was 46 (IQR 36 – 51 years). The sample comprised 18 Caucasian, 18 African American, 7 Native American, 7 Hispanic, and 3 Asian patients. The median time between visits was 1.8 months (IQR 1.1–3.3 months). Disease activity data are shown in Table 1. Patients with active disease in this routine lupus clinic setting most often had arthritis or mucocutaneous manifestations. Arthritis and rash as defined by the SLEDAI were present in 41 (77.4 %) and 14 (26.4 %) patients, respectively, at baseline. Thirty (56.6 %) and thirteen (24.5 %) patients, respectively, had A or B scores on the BILAG musculoskeletal and/or mucocutaneous domains at baseline. At baseline, 21 (39.6 %) patients had low C3 and/or C4 complement levels, and 10 (18.8 %) had positive results for anti-double-stranded DNA (anti-dsDNA) antibodies by *Crithidia luciliae* assay. Most patients were receiving hydroxychloroquine (Table 1), and about one-fourth were receiving chronic corticosteroids. Azathioprine, methotrexate, or mycophenolate mofetil was prescribed in 15–28 % of the patients, alone or in combination with hydroxychloroquine and prednisone.

**Table 1:** Characteristics of the study population at baseline and follow-up

Number of patients	Baseline (*n* = 53)	Follow-up (*n* = 53)	*p* Values
Disease activity, median (IQR)			
SLEDAI	6 (4–9)	4 (2–6)	**<0.001**
BILAG	9 (8–16)	2 (1–15)	**<0.001**
PGA	1.5 (1.2–1.8)	0.9 (0.6–1.4)	**<0.001**
Medications, *n* (%)			
HCQ	35 (66)	36 (68)	1.000
AZA	9 (17)	8 (15)	1.000
MTX	10 (19)	15 (28)	0.360
MMF	11 (21)	10 (19)	1.000
CS	13 (25)	12 (23)	1.000
Parameters affecting HRV, *n* (%)			
Tobacco exposure	14 (26)	16 (30)	0.829
Caffeine	27 (51)	30 (57)	0.697
Medications ↑HRV	12 (23)	11 (21)	1.000
Medications ↓HRV	13 (25)	11 (21)	0.816

*Abbreviations*: *SLEDAI* Systemic Lupus Erythematosus Disease Activity Index, *BILAG* British Isles Lupus Assessment Group, *PGA* physician global assessment, *HCQ* hydroxychloroquine, *AZA* azathioprine, *MTX* methotrexate, *MMF* mycophenolate mofetil, *CS* corticosteroids

P values < 0.05 are indicated in bold type

Parameters of HRV did not significantly change between baseline and follow-up (see Table S2 in Additional file 1). Exercise and exposure to tobacco, caffeine, and alcohol were similar between visits. Few patients had comorbidities known to affect HRV, including two patients with heart failure, two with recent seizures, one with untreated depression, one with diabetes, and one with asthma not controlled while on medications. Similar results were obtained when these seven individuals were omitted from the analysis; therefore, they were included in the final results. Exposure to medications known to increase HRV (β-blockers, calcium channel blockers, and clonidine) or decrease HRV (tricyclic antidepressants, antihistamines, β-agonists) did not differ between baseline and follow-up (Table 1). At baseline, seven patients were taking at least one medication known to increase HRV, eight were taking at least one known to decrease HRV, and five were taking combinations from both groups.

### HRV is inversely associated with disease activity and flare at baseline

**Fig. 1:**
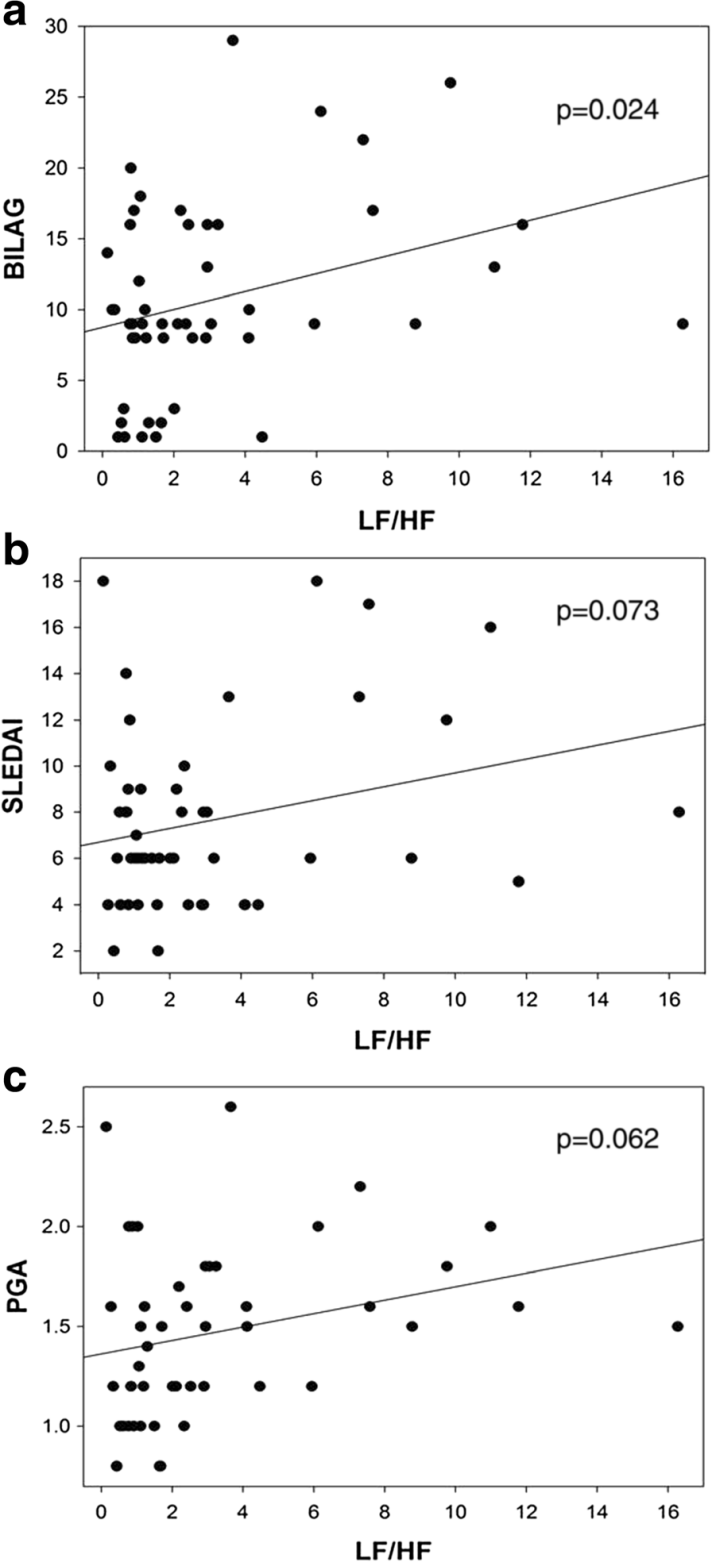
Association of baseline disease activity with sympathovagal balance measured as the low frequency to high frequency (LF/HF) ratio by univariate linear regression. **a** The British Isles Lupus Assessment Group (BILAG) index is inversely related to the LF/HF ratio (*p* = 0.024). Inverse trends were observed for **b** Systemic Lupus Erythematosus Disease Activity Index (SLEDAI) (*p* = 0.073) and **c** physician global assessment (PGA) (*p* = 0.062)

Relationships of HRV with disease activity were examined at baseline by univariate linear regression (Table 2). Total BILAG disease activity scores were inversely related to HRV parameters [pNN50 (*p* = 0.019), HF power (*p* = 0.020), LF/HF (*p* = 0.024)], and similar associations were observed when we focused on the most commonly involved organ, the musculoskeletal system [pNN50 (*p* = 0.043), HF power (*p* = 0.010)]. Total SLEDAI scores and arthritis by SLEDAI had a similar trend of association with HRV [LF/HF (*p* = 0.073 for total SLEDAI), HF power (*p* = 0.088 for SLEDAI arthritis)]. HRV was not associated with the presence of classic serological markers such as anti-dsDNA antibodies and low complement levels. The LF/HF ratio was associated with disease activity by BILAG (*p* = 0.024) and SFI (*p* = 0.008), although a trend was also seen in SLEDAI (*p* = 0.073) and PGA (*p* = 0.062) (Fig. 1). No association of HRV with tobacco or caffeine exposure or medications affecting autonomic nervous system function was demonstrable in this analysis. At the follow-up visits, no association of HRV with disease activity was observed.

**Table 2:** Association of disease activity with HRV parameters at baseline by univariate linear regression

	Independent variables			
Dependent variables	RMSSD	pNN50	HF	LF/HF
BILAG	*p* = 0.259 β = −0.024	***p*** ** = 0.019** β = −0.385	***p*** ** = 0.020** β = −0.108	***p*** ** = 0.024** β = 0.630
BILAG mucocutaneous	*p* = 0.870 β = 0.002	*p* = 0.124 β = −0.142	*p* = 0.313 β = −0.027	*p* = 0.695 β = 0.062
BILAG musculoskeletal	*p* = 0.346 β = −0.011	***p*** ** = 0.043** β = −0.185	***p*** ** = 0.010** β = −0.066	*p* = 0.100 β = 0.257
SLEDAI	*p* = 0.683 β = 0.005	*p* = 0.312 β = −0.101	*p* = 0.488 β = −0.020	*p* = 0.073 β = 0.301
SLEDAI rash	*p* = 0.977 β < 0.001	*p* = 0.420 β = −0.009	*p* = 0.170 β = −0.004	*p* = 0.206 β = 0.024
SLEDAI arthritis	*p* = 0.531 β < 0.001	*p* = 0.309 β = −0.010	*p* = 0.088 β = −0.005	*p* = 0.446 β = 0.013
PGA	*p* = 0.651 β < −0.001	***p*** ** = 0.014** β = −0.026	*p* = 0.154 β = −0.004	*p* = 0.062 β = 0.034
SFI	*p* = 0.456 β = −0.001	*p* = 0.329 β = −0.012	***p*** ** = 0.047** β = −0.007	***p*** ** = 0.008** β = 0.054

*Abbreviations: BILAG* British Isles Lupus Assessment Group, *HF* high-frequency power, *LF/HF* ratio low frequency to high frequency ratio, *PGA* physician global assessment, *pNN50* percentage of pairs of adjacent NN intervals differing by more than 50 milliseconds, *RMSSD* square root of the mean of the squares of differences between adjacent NN intervals, *SFI* Safety of Estrogens in Lupus Erythematosus National Assessment-SLEDAI flare index, *SLEDAI* Systemic Lupus Erythematosus Disease Activity Index

*p* values <0.05 are in bold type

### HRV is inversely related to soluble TNFRII and to IFN-induced cytokines

**Fig. 2:**
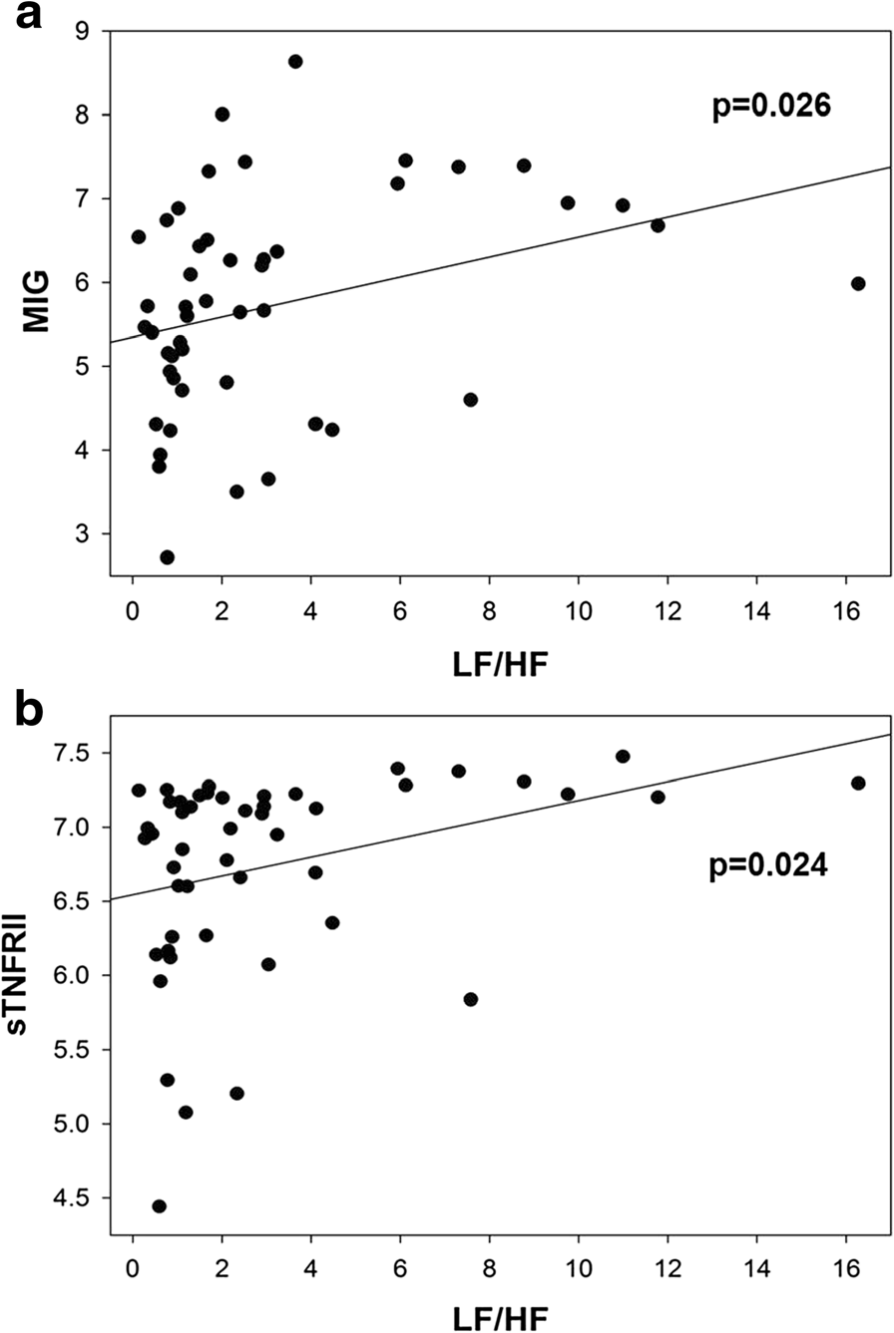
Association of plasma monokine induced by interferon gamma (MIG) and tumor necrosis factor receptor II (TNFRII) at baseline with the low frequency to high frequency (LF/HF) ratio by univariate linear regression. **a** MIG (*p* = 0.026) and **b** soluble TNFRII (sTNFRII) (*p* = 0.024) are both inversely related to the LF/HF ratio

Exploration of associations between HRV and cytokine levels at baseline was performed using linear regression without adjustment for multiple comparisons (Table 3). Elevated plasma sTNFRII and MIG were consistently associated with decreased HRV across all parameters measured (Table 3 and Fig. 2). Inverse associations with one or more parameters of HRV were also observed for plasma IL-1RA, SCF, and IL-15 (Table 3). Trends of similar associations were noted for IFN-α, IP-10 and serum BLyS (*p* < 0.10). There was no evidence to suggest that plasma IFN-γ, IL-10, IL-12, IL-1β, IL-1α, IL-2, IL-8, IL-17A, IL-21, IL-23, MIP-1α, sCD40L, TNFα, sTNFRI, PAI-1, resistin, TGF-β, or serum APRIL were related to HRV.

**Table 3:** Association of plasma cytokines with HRV parameters at baseline by univariate linear regression

	Independent variables			
Dependent variables	RMSSD	pNN50	HF	LF/HF
IL-1RA	*p* = 0.949 β < −0.001	*p* = 0.286 β = −0.069	***p*** ** = 0.011** β = −0.037	*p* = 0.067 β = 0.147
SCF	*p* = 0.133 β = −0.004	***p*** ** = 0.049** β = −0.042	*p* = 0.373 β = −0.005	***p*** ** = 0.034** β = 0.076
IL-15	*p* = 0.402 β = −0.003	*p* = 0.054 β = −0.054	***p*** ** = 0.025** β = −0.018	*p* = 0.148 β = 0.069
IFN-α	*p* = 0.920 β < −0.001	*p* = 0.089 β = −0.079	*p* = 0.063 β = −0.019	*p* = 0.313 β = 0.059
IP-10	*p* = 0.202 β = −0.005	*p* = 0.088 β = −0.048	*p* = 0.122 β = −0.012	*p* = 0.189 β = 0.064
MIG	***p*** ** = 0.007** β = −0.011	***p*** ** = 0.015** β = −0.077	***p*** ** = 0.018** β = −0.021	***p*** ** = 0.026** β = 0.119
sTNFRII	***p*** ** < 0.001** β = −0.007	***p*** ** = 0.01** β = −0.042	***p*** ** = 0.039** β = −0.010	***p*** ** = 0.024** β = 0.064
BLyS^a^	*p* = 0.556 β = 0.002	*p* = 0.201 β = −0.026	*p* = 0.140 β = −0.008	*p* = 0.098 β = 0.055

*Abbreviations: BLyS* B lymphocyte stimulator, *HF* high-frequency power, *IL* interleukin, *IL-1RA* interleukin 1 receptor antagonist, *IP-10* interferon gamma-induced protein 10, *LF/HF* low frequency to high frequency ratio, *MIG* monokine induced by interferon gamma, *pNN50* percentage of pairs of adjacent NN intervals differing by more than 50 milliseconds, *RMSSD* square root of the mean of the squares of differences between adjacent NN intervals, *SCF* stem cell factor, *sTNFRII* soluble tumor necrosis factor receptor II

^a^Serum BLyS

*p* values <0.05 are in bold type

Considering the potentially cyclical interplay between HRV and cytokines [10], we further explored which inflammatory pathway is most closely linked as a potential predictor of HRV in a multivariate model including plasma IL-1RA, SCF, IFN-α, IP-10, and serum BLyS. Associations of HRV with plasma MIG or sTNFRII remained significant (MIG: RMSSD *p* = 0.036, HF power *p* < 0.001, LF/HF *p* = 0.003; sTNFRII: RMSSD *p* = 0.011, HF power *p* = 0.040) (Table 4). In a correlation matrix of all parameters included in the models, there was a strong relationship between sTNFRII and MIG (Spearman’s rank correlation *R* = 0.751, *p* < 0.001); thus, these were not combined in the same model. Collinearity was also evident between sTNFRII and SCF (*R* = 0.508, *p* < 0.001), between MIG and IP-10 (*R* = 0.585, *p* < 0.001), and between MIG and SCF (*R* = 0.462, *p* < 0.001), as well as between IP-10 and IL-1RA (*R* = 0.564, *p* = 0.001), whereas all other *R* values were <0.4 (Table S3 in Additional file 1). Plasma sTNFRII was weakly correlated with plasma TNF-α (*R* = 0.357, *p* = 0.009) and sTNFRI levels (*R* = 0.273, *p* = 0.048).

**Table 4:** Multivariate linear regression of each heart rate variability parameter (dependent variable) with plasma cytokines (independent variables) at baseline

Independent variables	RMSSD		pNN50		HF		LF/HF	
	*p* Value	β coefficient	*p* Value	β coefficient	*p* Value	β coefficient	*p* Value	β coefficient
Model I								
IL-1RA	0.523	5.701	0.968	0.030	**<0.001**	−10.414	**0.009**	1.772
SCF	0.635	7.867	0.381	1.262	**0.034**	10.300	0.520	0.730
IFN-α	0.895	1.063	0.392	−0.602	0.739	−0.736	0.854	0.101
IP-10	0.762	5.658	0.725	0.567	**0.001**	19.838	**0.005**	−4.126
MIG	**0.036**	−34.531	0.072	−2.498	**<0.001**	−21.391	**0.003**	3.526
BLyS^a^	0.598	8.028	0.411	−1.087	**0.023**	−10.276	0.110	1.724
Model II								
IL-1RA	0.474	5.922	0.893	0.102	**0.018**	−8.913	**0.039**	1.555
SCF	0.559	9.075	0.384	1.259	0.185	8.802	0.488	0.927
IFN-α	0.867	1.262	0.414	−0.576	0.915	−0.336	0.949	0.041
IP-10	0.530	−8.722	0.618	−0.640	0.249	6.810	0.095	−2.070
sTNFRII	**0.011**	−51.594	0.078	−3.116	**0.040**	−16.762	0.061	3.100
BLyS^a^	0.476	10.003	0.504	−0.867	0.229	−7.168	0.303	1.253

*Abbreviations: BLyS* B lymphocyte stimulator, *HF* high-frequency power, *IFN* interferon, *IL* interleukin, *IL-1RA* interleukin 1 receptor antagonist, *IP-10* interferon gamma-induced protein 10, *LF/HF* low frequency to high frequency ratio, *MIG* monokine induced by interferon gamma, *pNN50* percentage of pairs of adjacent NN intervals differing by more than 50 milliseconds, *RMSSD* square root of the mean of the squares of differences between adjacent NN intervals, *SCF* stem cell factor, *sTNFRII* soluble tumor necrosis factor receptor II

^a^Serum BLyS

*p* values <0.05 are in bold type

### Some cytokines associated with HRV are also associated with disease activity

We further examined associations of baseline disease activity with plasma and serum cytokines by univariate linear regression (see Table S4 in Additional file 1). Plasma MIG was related to BILAG (*p* = 0.017) and PGA (*p* = 0.024), but no association was observed for sTNFRII. Several other cytokines appeared to be potentially associated with one or more measures of disease activity, stressing the different pathways that might be related to disease activity in different subsets of patients with SLE, only some of which might have an impact on HRV.

### Changes in HRV between visits are associated with changes in SLEDAI

Changes in SLEDAI between visits showed a significant inverse association with changes in RMSSD (*p* = 0.007), and there was also a trend of association with changes in pNN50 (*p* = 0.094) (Table 5). Trends of association (*p* > 0.05) of BILAG and PGA with RMSSD and HF power were also observed.

**Table 5:** Association of changes in disease activity and plasma cytokines with changes in heart rate variability by univariate linear regression

		Independent variables			
Dependent variables		ΔRMSSD	ΔpNN50	ΔHF	ΔLF/HF
Changes in disease activity	ΔBILAG	*p* = 0.081 β = −0.036	*p* = 0.605 β = −0.109	*p* = 0.168 β = −0.075	*p* = 0.119 β = 0.542
	ΔSLEDAI	***p*** ** = 0.007** β = −0.023	*p* = 0.094 β = −0.144	*p* = 0.326 β = −0.023	*p* = 0.431 β = 0.116
	ΔPGA	*p* = 0.209 β = −0.002	*p* = 0.704 β = −0.006	*p* = 0.067 β = −0.007	*p* = 0.174 β = 0.034
Cytokine changes	ΔsTNFRII	*p* = 0.824 β < −0.001	*p* = 0.789 β = 0.011	*p* = 0.168 β = −0.014	***p*** ** = 0.021** β = 0.150
	ΔMIG	*p* = 0.453 β = −0.004	*p* = 0.963 β = −0.003	*p* = 0.176 β = −0.019	***p*** ** = 0.003** β = 0.253
	ΔBLyS^a^	*p* = 0.298 β = −0.002	*p* = 0.994 β < −0.001	***p*** ** = 0.036** β = −0.010	*p* = 0.057 β = 0.054
	ΔIFN-α	*p* = 0.901 β < 0.001	*p* = 0.526 β = 0.037	***p*** ** = 0.004** β = −0.050	***p*** ** = 0.012** β = 0.356
	ΔIL-1RA	*p* = 0.840 β = −0.003	*p* = 0.794 β = −0.044	*p* = 0.199 β = −0.055	***p*** ** = 0.023** β = 0.646
	ΔIP-10	*p* = 0.671 β = −0.001	*p* = 0.679 β = 0.014	*p* = 0.945 β < −0.001	*p* = 0.895 β = 0.008
	ΔSCF	*p* = 0.085 β = −0.004	*p* = 0.603 β = −0.011	*p* = 0.154 β = −0.008	***p*** ** = 0.001** β = 0.108
	ΔIL-15	*p* = 0.764 β = −0.001	*p* = 0.471 β = 0.033	***p*** ** = 0.012** β = −0.026	***p*** ** = 0.010** β = 0.154

*Abbreviations: BILAG* British Isles Lupus Assessment Group, *BLyS* B lymphocyte stimulator, HF high-frequency power, *IFN* interferon, *IL* interleukin, *IL-1RA* interleukin 1 receptor antagonist, *LF/HF* low frequency to high frequency ratio, *MIG* monokine induced by interferon gamma, *PGA* physician global assessment, *pNN50* percentage of pairs of adjacent NN intervals differing by more than 50 milliseconds, *RMSSD* square root of the mean of the squares of differences between adjacent NN intervals, *SCF* stem cell factor, *SLEDAI* Systemic Lupus Erythematosus Disease Activity Index, *sTNFR* soluble tumor necrosis factor receptor

^a^Serum BLyS

*p* values <0.05 are in bold type

### Changes in the LF/HF ratio are related to changes in cytokines

Using linear regression, we examined changes in HRV in relation to changes in those cytokines that had been inversely related to HRV at baseline (Table 5). Changes of the LF/HF ratio were correlated with similar changes across all of those cytokines, including sTNFRII (*p* = 0.021) and MIG (*p* = 0.003). Changes in HF power were inversely related to changes only in serum BLyS (*p* = 0.036), plasma IFN-α (*p* = 0.004), and IL-15 (*p* = 0.012).

## Discussion

Although autonomic dysregulation is well documented in SLE, much remains to be learned about its direct or indirect relationships to disease activity. Although abnormalities in cardiovascular reflex testing and HRV have been described in SLE previously, these did not correlate with disease activity measured by BILAG [3] or SLEDAI [4, 6, 8]. In the present study, we found potential relationships between HRV and disease activity measured by SLEDAI, BILAG, and PGA, as well as the presence or absence of flare (Table 2). Furthermore, HRV changes between visits (time domain analysis and HF power) were correlated with changes in disease activity across all indices (Table 5). At follow-up, disease activity was generally improved, and the range of disease was far narrower in the population (Table 1), deterring the determination of cross-sectional linear relationships at those visits. These results are consistent with the known anti-inflammatory properties of vagus signaling [24] and raise the possibility that HRV might be a noninvasive marker for SLE disease activity and improvement, at least in a relevant subset of patients.

To begin a process by which such patients could be identified, we explored the association of HRV with a large panel of cytokines that was previously found at our institution to be relevant to SLE [25] (arrayed in Table S1 in Additional file 1). HRV was examined not only as a predictor (Tables 3 and 5) but also as an outcome (Table 4) of peripheral inflammation. Although the exact relationship between these variables is not yet known, evidence in both directions supports the hypothesis of a cyclical relationship [10]. This was an exploratory study, which limits the conclusions that can be drawn. Given the exploratory nature of our study and our small sample size, we elected not to adjust for multiple comparisons, in order to be able to detect associations of modest strength [26]. Notably, several strong preliminary associations were observed that provide useful hypotheses for further testing. In analysis that was not adjusted for multiple comparisons or other covariates, all HRV measurements were predictive of plasma MIG, an IFN type II-related cytokine, and sTNFRII, whose shedding has been associated with lupus flares [27] (Tables 3 and 5). HRV was also predictive of plasma SCF, a major macrophage growth and differentiation factor [28], while consistent inverse relationships with HRV were observed for two other IFN-related cytokines, IP-10 and IFNα, as well as for IL-1RA and serum BLyS (Tables 3 and 5). Although no relationship of HRV with plasma IFN-γ or TNF-α was noted in this analysis, our results support a potential association of vagal signaling with a Th1-mediated myeloid cell pathway, known to be important in subsets of patients with lupus [29]. The inverse association of HRV with plasma IL-15, a cytokine implicated in intestinal autoimmunity [30], is also intriguing because vagal signaling is known to reach myenteric neurons in close contact with intestinal macrophages [31]. Certain cytokines previously associated with HRV in experimental models of acute inflammation, such as TNF-α and IL-1β [24], were not impacted by HRV in this study of patients with lupus. Larger studies, however, might illuminate whether there is a subset of patients where these relationships could be observed or whether there is some reason why these are not linked to HRV or myeloid pathways in lupus. Cytokines can also differ between the systemic circulation and the tissue microenvironment, and autonomic regulation at the tissue level can be discordant with systemic autonomic effects. These considerations further limit the conclusions that can be drawn from this preliminary study.

Clinically evident arthritis was the most prevalent SLE manifestation in our cohort, and arthritis alone was associated with decreased parasympathetic activity. A direct effect of vagal signaling in the joints is unlikely; nonetheless, this finding might be clinically meaningful, considering the promising role of intermittent electrical vagal nerve stimulation in animal models of arthritis [32] and in patients with rheumatoid arthritis [33].

Multiple potentially confounding factors can affect HRV, including age, cardiovascular disease, diabetes, renal failure, obstructive sleep apnea, tobacco, caffeine, and drugs [34–36]. Although few patients in this study had such obvious features, cardiovascular disease and prediabetes are often subclinical in SLE [37, 38], so further evaluation of these potential confounders is in order. No association with smoking or caffeine consumption was evident, but more sensitive measures to record and quantify the degree of these exposures might have captured an effect. It is also possible that HRV is more sensitive to psychosocial factors than lupus disease activity itself, especially in patients with mild to moderate disease. We did not formally assess depression, pain, sleep disturbances, and fatigue, all of which are common in SLE and known to be associated with decreased HRV [39, 40]. A more comprehensive assessment of autonomic nervous system function by additional clinical cardiovascular reflex testing, as well as assessment of baroreflex sensitivity and respiratory sinus arrhythmia, might have also allowed better discernment of these effects.

Frequency domain parameters correlated better than time domain indices with disease activity and/or cytokines in this study. This is somewhat expected, as we used only 5-minute ECG recordings, and time domain analysis has higher variability with shorter durations [22]. In addition to the limitations of HRV analysis [41], those pertinent to the clinical measures of lupus activity should be acknowledged to prevent overinterpretation of these findings [42].

## Conclusions

Our results suggest that impaired HRV, particularly the LF/HF ratio, is associated with lupus disease activity and several cytokines related to IFN type II and TNF pathways. The cytokines with the strongest association were MIG and sTNFRII, confirming and expanding previous connections between vagal signaling and immunity. These data support a potentially important interplay between autonomic nervous system function and lupus disease activity, as well as a possible role of vagal modulation as adjunctive therapy in SLE. This can be approached by pharmacological or electrical treatments or through cognitive behavioral therapy approaches [24]. Such therapies are rapidly entering clinical research programs and may be applicable to SLE and other clinical inflammatory disorders.

## Additional file

Additional file 1:
**Table S1.** Plasma cytokines measured at each visit by multiplex bead-based immunoassay. **Table S2.** Parameters of HRV (RMSSD, pNN50, HF power, and the LF/HF ratio) at baseline and follow-up. HRV parameters are compared between visits by paired analyses (Wilcoxon signed-rank test or paired *t* test); *p* values are listed. **Table S3.** Matrix of Spearman’s rank correlations (*R*) between cytokines included in the multivariate models. *p* values <0.1 are included, and *R* values that are statistically significant (*p* < 0.05) are shown in bold type. **Table S4.** Associations of disease activity and flare with plasma cytokines at baseline by univariate linear regression (*p* values and beta regression coefficients). (DOCX 23 kb)

## Abbreviations

anti-dsDNA: anti-double-stranded DNA
APRIL: A Proliferation-Inducing Ligand
AZA: Azathioprine
BILAG: British Isles Lupus Assessment Group
BLyS: B Lymphocyte Stimulator
CS: Corticosteroids
ECG: Electrocardiogram
HCQ: Hydroxychloroquine
HF power: High-Frequency Power
HIPAA: Health Insurance Portability and Accountability Act
HRV: Heart Rate Variability
IFN: Interferon
IL: Interleukin
IL-1RA: Interleukin 1 Receptor Antagonist
IP-10: Interferon Gamma-Induced Protein 10
IQR: Interquartile range
IRB: Institutional Review Board
LF power: Low Frequency Power
LF/HF ratio: Low Frequency to High Frequency ratio
MIG: Monokine Induced by Interferon Gamma
MIP-1α: Macrophage Inflammatory Protein 1 Alpha
MMF: Mycophenolate Mofetil
MTX: Methotrexate
NN: Normal to Normal
OMRF: Oklahoma Medical Research Foundation
PAI-1: Plasminogen Activator Inhibitor 1
PGA: Physician Global Assessment
pNN50: Percentage of Pairs of Adjacent NN Intervals Differing by more than 50 Milliseconds
RMSSD: Square Root of the Mean of the Squares of Differences between Adjacent NN Intervals
SCF: Stem Cell Factor
SELENA: Safety of Estrogens in Lupus Erythematosus National Assessment
SFI: SELENA-SLEDAI Flare index
SLE: Systemic Lupus Erythematosus
SLEDAI: Systemic Lupus Erythematosus Disease Activity Index
sTNFR: soluble Tumor Necrosis Factor Receptor
TGF-β: Transforming Growth Factor Beta
TNF-α: Tumor Necrosis Factor Alpha
