# Supplementary material for: Impact of heart rate variability, a marker for cardiac health, on lupus disease activity
Source: Arthritis Res Ther. 2016 Sep 2;18(1):197. doi: 10.1186/s13075-016-1087-x (PMC5010705; doi:10.1186/s13075-016-1087-x)
Supplement: Additional file 1: — Table S1. Plasma cytokines measured at each visit by multiplex bead-based immunoassay. Table S2. Parameters of HRV (RMSSD, pNN50, HF power, and the LF/HF ratio) at baseline and follow-up. HRV parameters are compared between visits by paired analyses (Wilcoxon signed-rank test or paired t test); p values are listed. Table S3. Matrix of Spearman’s rank correlations (R) between cytokines included in the multivariate models. p values <0.1 are included, and R values that are statistically significant (p < 0.05) are shown in bold type. Table S4. Associations of disease activity and flare with plasma cytokines at baseline by univariate linear regression (p values and beta regression coefficients). (DOCX 23 kb) [file 13075_2016_1087_MOESM1_ESM.docx]

Table S1. Plasma cytokines measured at each visit by multiplex bead-based immunoassay

| **Th1-like** | **Th17-like** | **Regulatory** | **Other** |
| --- | --- | --- | --- |
| IFNγ | IL-17A | TGFβ | PAI-1 |
| IL-12p70 | IL-21 | IL-10 | SCF |
| IL-2 |  |  | Resistin |
| **Innate immunity** | **TNFR superfamily** | **Chemokines** |  |
| IL-1α | TNFα | IFNα |  |
| IL-1β | sTNFRI/II | IP-10 |  |
| IL-1RA | sCD40L | MIP-1α |  |
| IL-15 | BLyS | MIG |  |
|  | APRIL | IL-8 |  |

Table S2. Parameters of HRV at baseline and follow up. HRV parameters are compared between visits by paired analyses.

|  | **Baseline** | **Follow up** | **P values** |
| --- | --- | --- | --- |
| **Number of patients** | n = 49 | n = 41 | Paired  n = 39 |
| **HRV: medial (IQR)** |  |  |  |
| RMSSD | 22.0 (12.2 - 42) | 26.7 (13 - 53.2) | 0.994* |
| pNN50 | 0.5 (0 - 2.6) | 3 (0 – 2.0) | 0.615* |
| HF | 37.0 (20.6 - 53.7) | 31.0 (20.9 - 48.7) | 0.076** |
| LF/HF ratio | 1.7 (0.9 - 3.9) | 2.2 (1.1 - 3.9) | 0.197* |

* Wilcoxon Signed Rank test, ** Paired t-test

Table S3. Martix of Sperman Rank correlations (R) between cytokines included in the multivariate models. P values <0.1 are included and R values that are statistically significant (p<0.05) are in bold.

| **R** | **BLyS** | **IFNα** | **IL-1RA** | **IP-10** | **SCF** | **MIG** |
| --- | --- | --- | --- | --- | --- | --- |
| **IFNα** | 0.203 |  |  |  |  |  |
| **IL-1RA** | -0.0598 | 0.339 |  |  |  |  |
| **IP-10** | **0.342**  p=0.016 | **0.390**  p=0.013 | **0.564**  p=0.001 |  |  |  |
| **SCF** | 0.272  p=0.059 | 0.213 | 0.250 | 0.233  p=0.092 |  |  |
| **MIG** | 0.257  p=0.075 | 0*.*294  p=0.066 | 0.303 | **0.585**  p<0.001 | **0.462**  p<0.001 |  |
| **sTNFRII** | 0.259  p=0.073 | 0.263 | 0.223 | **0.392**  p=0.004 | **0.508**  p<0.001 | **0.751**  p<0.001 |

Table S4. Associations of disease activity and flare with plasma cytokines at baseline by univariate linear regression (p values and regression coefficients β).

|  | **Dependent variables** | | | |
| --- | --- | --- | --- | --- |
| **Independent variables** | **BILAG** | **SLEDAI** | **PGA** | **SFI** |
| **IL-10** | p=0.122  β=1.145 | p=0.191  β=0.640 | p=0.082  β=0.0837 | p=0.978  β=-0.002 |
| **IL-1α** | p=0.066  β=1.183 | p=**0.004**  β=1.059 | p=**0.018**  β=0.096 | p=0.533  β=0.031 |
| **IL-1RA** | p**<0.001**  β=2.984 | p=**0.013**  β=1.139 | p=**0.013**  β=0.129 | p=0.053  β=0.103 |
| **IL-1RA/IL-1β** | p=**0.016**  β=2.495 | p=0.119  β= 0.970 | p=0.260  β= 0.080 | p=0.900  β= 0.009 |
| **IL-8** | p=**0.015**  β=2.796 | p=0.173  β=1.019 | p=0.232  β=0.094 | p=0.089  β=0.134 |
| **IL-21** | p=0.147  β=1.051 | p=**0.035**  β=0.838 | p=**0.035**  β=0.098 | p=0.764  β=-0.016 |
| **IL-23** | p=0.136  β=1.750 | p=**0.041**  β=1.486 | p=0.269  β=0.086 | p=0.244  β=0.105 |
| **INFα** | p=0.116  β=1.288 | p=**0.014**  β=1.303 | p=**0.017**  β=0.129 | p=0.873  β=-0.010 |
| **IP-10** | p=**0.001**  β=2.460 | p=**0.002**  β=1.514 | p=**0.002**  β=0.160 | p=**0.020**  β=0.141 |
| **MIG** | p=**0.017**  β=1.693 | p=0.259  β=0.505 | p=**0.024**  β=0.106 | p=0.190  β=0.073 |
| **sCD40L** | p=0.811  β=-0.242 | p=0.099  β=-1.019 | p=0.669  β=-0.029 | p=0.977  β=-0.002 |
| **TNFα** | p=0.099  β=1.882 | p=0.699  β=-0.277 | p=0.516  β=0.049 | p=0.810  β=-0.021 |
| **sTNFRI** | p=0.797  β=0.260 | p=0.251  β=0.715 | p=0.085  β=0.114 | p=0.338  β=0.075 |
| **APRIL** | p=**0.035**  β=2.213 | p=**0.009**  β=1.650 | p=**0.012**  β=0.170 | p=0.393  β=0.070 |
| **Resistin** | p=0.241  β=2.193 | p=**0.035**  β=2.401 | p=0.122  β=0.189 | p=0.623  β=0.071 |

p values <0.05 are in bold type
